# Trends of Hepatitis A Virus Infection in Poland: Assessing the Potential Impact of the COVID-19 Pandemic and War in Ukraine

**DOI:** 10.3390/v16030469

**Published:** 2024-03-20

**Authors:** Piotr Rzymski, Dorota Zarębska-Michaluk, Agnieszka Genowska, Piotr Tyszko, Birute Strukcinskiene, Robert Flisiak

**Affiliations:** 1 Department of Environmental Medicine, Poznań University of Medical Sciences, 60-806 Poznań, Poland; 2 Department of Infectious Diseases and Allergology, Jan Kochanowski University, 25-317 Kielce, Poland; dorota1010@tlen.pl; 3 Department of Public Health, Medical University of Bialystok, 15-295 Bialystok, Poland; agnieszka.genowska@umb.edu.pl; 4 Department of Social Medicine and Public Health, Medical University of Warsaw, 02-091 Warsaw, Poland; piotr.tyszko@wum.edu.pl; 5 Institute of Rural Health, 20-090 Lublin, Poland; 6 Faculty of Health Sciences, Klaipeda University, LT-92294 Klaipeda, Lithuania; birute.strukcinskiene@ku.lt; 7 Department of Infectious Diseases and Hepatology, Medical University of Bialystok, 15-540 Bialystok, Poland

**Keywords:** viral hepatitis, epidemiology, COVID-19 pandemic, war refugees, Central Europe

## Abstract

Hepatitis A virus (HAV) is the most common cause of acute viral hepatitis, which is preventable by vaccination. This study analyzed trends of HAV infections in Poland according to socio-demographic features in the years 2009–2022 and assessed the potential impact of the COVID-19 pandemic (2020–2023) and the migration of war refugees from Ukraine (since February 2022). In 2009–2022, 7115 new cases of HAV infection were diagnosed in Poland, especially among men (66.4%) and in urban areas (77.4%). Infections among men were most common at the age of 25–34 (median rate 0.43 per 10^5^) and in women aged 15–24 (median rate 0.39 per 10^5^). Analysis of the 14-year frequency of HAV infections exhibited three trends, regardless of gender, age, and residence. The infections revealed a downward trend in 2009–2014, increased significantly in 2014–2018, and decreased again after 2018. A particularly rapid increase in HAV infections occurred between March 2017 and February 2018 (median rate 0.79 per 10^5^). The high level of new infections persisted until the beginning of the COVID-19 pandemic, at which point it dropped significantly but did not reach the level recorded before March 2017. During the Omicron SARS-CoV-2 dominance period, the median rate of HAV infections was 0.053 per 10^5^, with a four-fold increase being observed from February 2022 (when the migration of war refugees from Ukraine began) to August 2022. The presented results can serve as a reference point for further observations in Central Europe. The HAV epidemiological situation is unlikely to escalate in Poland but requires further monitoring.

## 1. Introduction

Hepatitis A is caused by a nonenveloped, positive-sense, single-stranded RNA virus (HAV) with a 7.5 kb genome, represented by one serotype and seven genotypes [1]. It is globally prevalent and remains the most common form of acute viral hepatitis worldwide [2]. Humans, its only natural host (though some nonhuman primates have been experimentally infected), are infected predominantly through the oral-fecal route due to the consumption of contaminated food and water, but can also acquire infection through close physical contact with an infected individual, mainly during sexual and anal intercourse [3]. A blood-borne route of transmission, e.g., through intravenous drug use, is possible, although rarely [4]. According to data provided by the World Health Organization (WHO), up to 1.5 million HAV infections are reported annually; this figure refers only to symptomatic cases, thus not fully reflecting the scale of the phenomenon [2,5]. It is estimated that their actual number may be several-fold higher, with some assessments indicating that over 150 million infections can occur annually. Such a high number is plausible because the clinical course of infection is mostly asymptomatic in highly endemic countries where HAV is acquired in childhood [6,7].

The high prevalence of HAV infections is closely related to poor sanitary and hygienic conditions and lack of access to safe and good-quality drinking water, meaning that highly endemic regions include developing countries, while in developed areas, the infection rates are low [8]. However, in recent years, an unfavorable trend has been outlined, with hepatitis A continuing to occur in hyperendemic regions and emerging in areas of low endemicity [7]. Not only are improvements in socioeconomic, sanitary, and hygienic conditions contributing to changes in HAV epidemiology, but it is also influenced by increased economic interdependence and social integration, along with changing human behavior and interactions, including those in the sexual sphere, the globalization of trade, the increasing pace of international travel, and migration movements [9]. Many adults in low-prevalence countries are susceptible to HAV infection, which, in the face of increasing social integration, intensified travel, and trade that crosses national borders, poses a high risk of compensatory outbreaks. At particular risk of HAV infection are people traveling to highly endemic areas, child care workers, men who have sex with men (MSM), users of illegal drugs, persons living in isolated communities including jails, those experiencing homelessness, migrants and refugees, and people with occupational exposure, such as healthcare workers and persons working with food and municipal waste [8,10]. Groups with a high risk of severe consequences due to hepatitis A include people living with HIV/AIDS and patients with chronic hepatitis [10].

Prevention efforts in the form of vaccinations, which have been available since the early 1990s, should focus on those populations at high risk of HAV infection in low-endemic areas [5,11]. Although acute symptomatic hepatitis A is generally a mild self-limiting disease with a low risk of fulminant hepatitis and death, patients with chronic liver disease are at risk of a more severe course and should also be vaccinated [12]. However, it should be noted that HAV vaccination in many low-endemic countries is not mandatory but is only recommended, so despite the availability of this form of prophylaxis, its impact in practice depends on the implementation of guidelines [4,13].

Over the past few decades, the most significant impact on the epidemiology of HAV infections in Poland has been due to improvements in socioeconomic and sanitary-hygienic conditions, which led to a transition from the high endemicity category in the 1970s and 1980s to low endemicity in the early 2000s [14]. However, there is a lack of studies summarizing trends in HAV epidemiology that have taken place in Poland in recent years in the face of the globalization phenomena, the COVID-19 pandemic, and the inflow of war refugees from Ukraine [15]. Therefore, there is a pivotal need to fill this knowledge gap in order to assess the effectiveness of prevention measures. To this end, the present study aimed to track HAV epidemiological trends in Poland from 2009 to 2022, taking into account place of residence, age, and gender, along with the identification and characterization of epidemic outbreaks and an assessment of the potential impact of the COVID-19 pandemic and migratory movements related to the war in Ukraine, which began in February 2022.

## 2. Materials and Methods

### 2.1. Study Design

In this retrospective study in Poland, we analyzed all registered cases of HAV infection over the 14 years from 2009 to 2022. The starting period for data collection was set at 2009, considering comparable data on HAV, which was presented according to a uniform methodology, and covered each subsequent calendar year until 2022 (as the last year in which complete data on infections were available) [16]. The study included information on newly diagnosed cases of HAV infection, along with socio-demographic characteristics, such as gender, age, and place of residence. To estimate the potential impacts of the COVID-19 pandemic and war in Ukraine, we analyzed new HAV infections in the following periods: pre-pandemic (March 2019 to February 2020), the COVID-19 period (March 2020 to May 2023), and the period during the migration of the war refugees from Ukraine (February 2022 to December 2023). Our epidemiological analysis was based on secondary administrative data from a publicly accessible national registry; therefore, obtaining approval from a biomedical ethics committee was not required.

### 2.2. Data Sources and HAV Infection Variables

Information on diagnosed HAV infection was obtained from the Epimeld database, published by the National Institute of Public Health-National Research Institute (NIPH-NRI) and the Chief Sanitary Inspectorate in Poland [17,18]. The analysis included newly diagnosed HAV infections that were identified using the 10th revision of the International Statistical Classification of Diseases and Related Health Problems (ICD-10), with codes for HAV infection cases (B15, including B15.0 and B15.9). The rate of newly diagnosed HAV cases was calculated as the number of new cases per 100,000 population in each calendar year and considered the division into age groups (≤14, 15–24, 25–34, 35–44, 45–54, and ≥55), gender, and place of residence (urban and rural).

The potential impact of the COVID-19 pandemic phases on HAV epidemiology was investigated using monthly anonymous data from March 2020 to December 2023 by comparison with pre-pandemic data, i.e., seven calendar years before the announcement of the epidemic, from March 2013 to February 2020. The study also included newly diagnosed HAV cases in the period of migration of the war refugees from Ukraine from February 2022 to December 2023, in relation to the pre-pandemic period. Monthly data on recorded newly diagnosed HAV cases from March 2019 to December 2023 was obtained from epidemiological reports on infectious diseases published regularly by the NIPH-NRI [18].

To assess the burden of newly diagnosed HAV cases during the COVID-19 pandemic, we explored the pre-pandemic period, depending on the dominant variants of SARS-CoV-2 [19,20,21]. The pandemic period was divided into four periods: (i) the pre-Delta lineage period from March 2020 to June 2021, (ii) the Delta lineage dominance period from July 2021 to December 2021, (iii) the early phase of the Omicron SARS-CoV-2 lineage dominance from January 2022 to June 2022 (dominated by the BA.1 and BA.2 Omicron subvariants), and (iv) a later phase of Omicron SARS-CoV-2 dominance from July 2022 to December 2023 (characterized predominantly by the dominance of XBB and its descendent sublineage). These periods were established based on genomic viral sequences that were submitted by Polish laboratories according to the Global Initiative on Sharing All Influenza Data (GISAID), the database on the prevalence of SARS-CoV-2 (sub)variants in different regions of the world [22].

In order to assess the influence of the migration of war refugees from Ukraine on the epidemiology of HAV in Poland, we analyzed rates of newly diagnosed HAV cases in the corresponding months from February 2022 (when the Ukrainian–Russian war began) to December 2023. The rate of HAV diagnoses was calculated as the monthly number of new cases per 100,000 population.

### 2.3. Statistical Analyses

Categorical variables were presented as proportions and compared using a chi-square test. A two-proportion test with Bonferroni correction for multiple comparisons was also implemented to compare newly diagnosed HAV cases between individual pairs of subgroups. Wilcoxon’s signed-rank test was used to compare the distributions of HAV diagnosis rates between rural and urban settings. In order to explore the long-term trends in new HAV infections during the 2009–2022 period, joinpoint regression was performed to examine changes in the trends [23,24]. We started with the minimum number of joinpoints (e.g., zero joinpoints, which is a straight line) and used a maximum of two joinpoints (corresponding to three-line segments). An annual percentage change (APC) with a corresponding 95% confidence interval was calculated for each linear segment. In addition, the average annual percentage change (AAPC) and its 95% CI in the trends for the period between 2009 and 2022 were also calculated [25]. The burden of hepatitis A during the COVID-19 pandemic and the migration of war refugees from Ukraine was analyzed by calculating the percentage change in the rates of newly diagnosed HAV cases. All statistical calculations were conducted using the IBM^®^ SPSS^®^ Statistics for Windows, version 24.0 statistical package (IBM Corporation, Armonk, NY, USA); a *p*-value of < 0.05 was considered statistically significant.

## 3. Results

### 3.1. General Epidemiology of HAV

Between 2009 and 2022, a total of 7115 new cases of HAV infection were diagnosed in Poland, with a higher proportion found in men (66.4% of diagnosed cases, *n* = 4724). Of the total newly diagnosed cases of HAV, a significant proportion was in urban areas (77.4%). Differences in newly diagnosed HAV cases by gender in urban and rural areas were statistically significant (*p* ≤ 0.001). Table 1 presents the descriptive statistics for the newly diagnosed HAV infection rate distribution. In 2009–2022, the median rate for newly diagnosed HAV infection among men was 0.30 per 10^5^, which was higher compared to women, at 0.24 per 10^5^ (*p* ≤ 0.001). The lowest median of newly diagnosed HAV infection was observed in the age group of ≥55 years old, among men at 0.19 per 10^5^, and among women at 0.12 per 10^5^ (*p* < 0.05). Median HAV infection rates were also low in men aged 45–54 (0.20 per 10^5^). The highest rates of newly diagnosed HVA infection were found in men aged 25–34 years old (0.43 per 10^5^) and in women in the age groups of 15–24 and 25–34 (0.39 per 10^5^ and 0.37 per 10^5^, respectively). In the analyzed period of 2009–2022, the median total rate for newly diagnosed HAV infection among men inhabiting urban areas was two-fold higher compared to rural areas (0.39 per 10^5^ vs. 0.21 per 10^5^; *p* ≤ 0.002). Similarly, a higher rate was noted for women living in urban areas than in rural regions (0.29 per 10^5^ vs. 0.14 per 10^5^, respectively; *p* ≤ 0.01) (Table 1).

### 3.2. Joinpoint Analysis of Long-Term Trends in New HAV Infections

**Figure 1:**
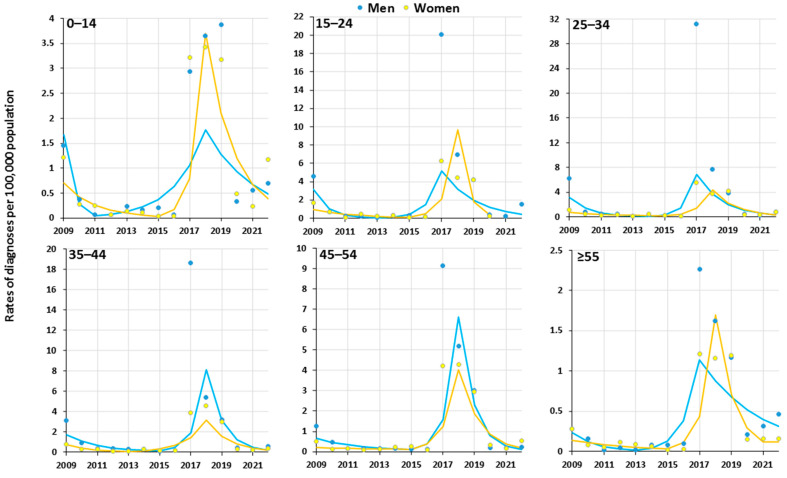
Hepatitis A virus infection in Poland over the years 2009–2022 as a trend, modeled with joinpoint regression.

The results of the joinpoint analysis of the 14-year trends of newly diagnosed HAV rates are presented in Table 2 and Figure 1. They indicate changes in the trend direction of HAV infections from 2009 to 2022 when they appeared in two joinpoints connecting a three-line segment of the trend. Regardless of gender, age, and place of residence, the first trend was decreasing, while in the second segment of the trend, the direction was reversed to positive, with high APC values (in men, from +68.3% to +388.1%; in women, from 119.5% to +374.7%). Eventually, the third trend line was downward. Among men in the first period in 2009–2014, the total rate of newly diagnosed HAV infection decreased from 2.69 per 10^5^ to 0.20 per 10^5^ (APC_2009–2014_ −47.4%, *p*_trend_ < 0.05). In subsequent years (trend 2), the rate showed a sharp increase up to 12.55 per 10^5^ (APC_2014–2017_ +312.2%, *p*_trend_ > 0.05). In the third segment, the total rate for newly diagnosed HAV infection decreased to 0.64 per 10^5^ (APC_2017–2022_ −39.2%, *p*_trend_ > 0.05). In the analysis by age, in the age groups of ≤ 14, 15–24, and ≥ 55 among men over the 2009–2022 period, none of the three-line segments was significant (*p*_trend_ > 0.05). However, in the age group of 25–34 among men in the initial period of 2009–2014 (trend 1), the newly diagnosed HAV infection rate decreased (APC_2009–2014_ −54.9%, *p*_trend_ < 0.05); the second and third line segments showed no significant changes (*p*_trend_ > 0.05). The trend was downward in the age group of 35–44 among men in the first period (APC_2009–2015_ −37.3%, *p*_trend_ <0.05). In the second trend, there was an increase that did not reach statistical significance, while in the third trend after 2018, there was a pronounced decreasing trend (APC_2018–2022_ −60.9%, *p*_trend_ < 0.05). In the age group of ≥ 55, significant changes were revealed in the third segment of the trend (APC_2018–2022_ −65.3%, *p*_trend_ < 0.05). Among women, the trends were similar to those observed in men but did not reach statistical significance. The total rate for newly diagnosed HAV infection among women in the first segment of the trend decreased from 0.80 per 10^5^ to 0.11 per 10^5^ (APC_2009–2015_ −25.1%, *p*_trend_ > 0.05); then, in the trend for period 2, there was rapid growth (APC_2015–2018_ +237.3%, *p*_trend_ > 0.05), and the third trend was downward (APC_2018–2022_ −49.1%, *p*_trend_ > 0.05). A significant decrease was noted only among urban men in the first trend (APC_2018–2022_ −65.3%, *p*_trend_ < 0.05), while other trends related to place of residence among men and women were insignificant (Table 2).

### 3.3. HAV Infections during the COVID-19 Pandemic and Migration of War Refugees

**Figure 2:**
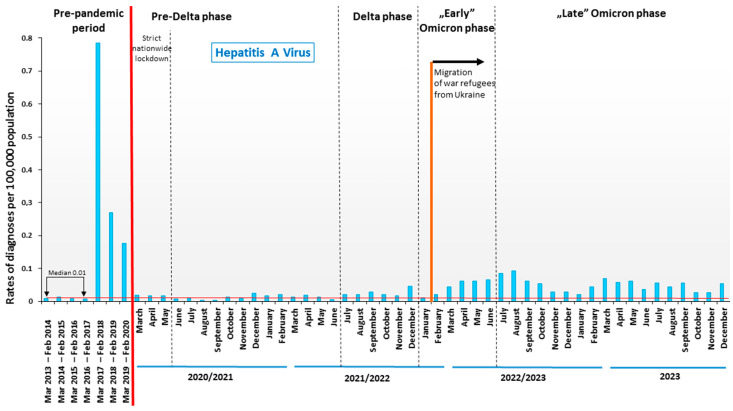
The burden of hepatitis A virus in Poland prior to and during the COVID-19 pandemic and the migration of war refugees from Ukraine.

In the pre-pandemic period, new HAV infections reached very low levels over a four-year period (from March 2013 to February 2017), during which the median was 0.01 per 10^5^. After this period, there was a sharp increase in infections related to the hepatitis A epidemic, and in the first 12 months from March 2017 to February 2018, the median number of new HAV infections increased to 0.79 per 10^5^. The high level of new HAV infections continued for another 24 months, up to February 2020 (Figure 2). When the COVID-19 pandemic occurred, the level of new HAV infections decreased significantly, but the value of newly diagnosed HAV rates did not reach the previous level before March 2017. During the 39 months of the COVID-19 pandemic (from March 2020 to May 2023), newly diagnosed HAV infection rates were generally higher than during the corresponding months in the pre-pandemic period from March 2013 to February 2017. In the pre-Delta phase, the median rate of newly diagnosed HAV was 0.013 per 10^5^ (by +30% vs. 0.01 per 10^5^ in the pre-pandemic period from March 2013 to February 2017). In the subsequent pandemic period (Delta phase), the median for new HAV diagnoses was 0.021 per 10^5^ (by +110% vs. the period from March 2013 to February 2017). A significant increase in new HAV infections occurred in the earlier Omicron phase, which coincided with the migration of war refugees from Ukraine. Specifically, from February 2022 (the month in which the Russian army invaded Ukraine) to August 2022, the rates of new HAV diagnoses systematically increased (from 0.021 to 0.093 per 10^5^). In the earlier and later Omicron phases, the median for new HAV diagnoses was 0.053 per 10^5^ (by +430% vs. the period from March 2013 to February 2017).

## 4. Discussion

The present study provides novel insights into the epidemiological situation of HAV infections in Poland, which are pivotal for shaping the policy on prevention measures and targeting specific groups. HAV vaccines, which are highly immunogenic and are effective in preventing infection when given as a two-dose series to children and adults [26,27], have been available in Poland since 1995 but are currently completely voluntary, resulting in low vaccine coverage (0.80 per 1000 inhabitants in 2020–2022 [16]), even among high-risk groups, e.g., MSM [28]. This is because Poland is currently considered a region of low endemicity [14]. However, the present study shows that temporary spikes in newly diagnosed HAV cases can occur, as evidenced especially for the 2014–2017 period, while the epidemiological situation may be significantly affected in both a downward and upward manner by novel circumstances, such as a pandemic of respiratory disease and war in a neighboring country. These findings highlight the need for the continuous monitoring of HAV in Poland and for potentially targeting specific groups with vaccination.

As shown, HAV infections were more common in men. This observation is in line with observations made previously in Poland [29], although there are reports of a slightly higher prevalence of hepatitis E in women in some countries [30]. Contrary to infections with the hepatitis E virus, which are often clinically indistinguishable from HAV infections, the latter are not more likely to present clinically in men compared to women [31], implying that other factors responsible for higher morbidity within males. Firstly, hepatitis A disproportionately affects MSM due to increased transmission risk, particularly during oro-anal and genito-oral sexual intercourse, as also shown when analyzing the multi-country outbreak that occurred in countries in the European Economic Area in 2016/2017 [32]. Secondly, independently from sexual behavior, the higher frequency in men may also result from worse hygiene habits in men, e.g., several studies have documented that men wash their hands less often than women, as also seen during the period of the COVID-19 pandemic, when frequent handwashing was particularly recommended by the health authorities [33,34,35]. In turn, handwashing, including only with tap water, is known to reduce HAV particles significantly and is considered one of the best prevention measures against direct and indirect spread [36].

The present study found that a spike in HAV infections occurred, regardless of sex, in individuals aged 15–44, especially in the 25–34-year-old group. This observation is likely a result of age-related increased sexual activities, including the exploration of sexual novelty [37]. Notably, there was no increase in HAV infections among children in 2018–2022, though their incidence was higher than in 2009–2013. HAV infections in children are concerning, due to the potential long-term health effects [38]. Despite low vaccination coverage in Poland, HAV circulation in this group remains low and is not expected to increase since socioeconomic and sanitary conditions are improving.

Our study also reports that HAV infections were consistently more common in individuals, both women and men, who inhabit urban areas. This is an interesting finding, especially if one considers that some epidemiological studies conducted in other world regions point to the contrary, implying that the urbanization process is generally associated with a reduction in hepatitis A morbidity due to comprehensive improvement in sanitary facilities related to water supply, excrement disposal, and environmental hygiene compared to rural areas [39]. Conversely, a study in Korea evidenced a higher prevalence of HAV infections in urban areas with a high population density [40], which is in line with our observations. This heterogeneity in the relationship between urbanization and hepatitis A epidemiology is likely due to different factors playing leading roles in HAV spread in a particular population. In developing countries, urbanization may curb the incidence of HAV due to improved access to clean water. In turn, in high-income areas, where discrepancies in this access are not so profound between urban and rural areas, the spread in adults may be driven by imported food and particular sexual behaviors, especially those typical of MSM, who tend to reside in or move to urban areas [9,41]. It is also plausible that individuals inhabiting urban areas in Poland have better access to healthcare services, including diagnostics, and may be more frequently tested for HAV infections, resulting in their higher identification. Therefore, the possibility cannot be excluded that the HAV prevalence reported in the present study for rural areas may be underestimated due to differences in access to medical care.

It is known that the COVID-19 pandemic has affected the epidemiology of various viral infections through two main pathways: (i) limited access to diagnostics as a result of the reorganization of the healthcare system and imposed lockdown measures, and (ii) attenuated transmissibility due to sanitary measures and social distancing [42,43]. We found that during the early phase of the pandemic and during the dominance of the Delta SARS-CoV-2 variant, the rate of newly diagnosed HAV infections was lower than in preceding years. In the pre-pandemic period, an increasing trend in hepatitis A notifications was generally noted in Europe, with clustered outbreaks in MSM noted in 2017 [44,45]. However, HAV infections in Poland were more frequent than in earlier periods between March 2013 and February 2017. This is an interesting finding since, according to the European Centre for Disease Prevention and Control, the hepatitis A notification rate during the pandemic, especially in 2021, was exceptionally low in the European Economy Area, a phenomenon attributed not only to the lockdowns, restrictions, and reduced international travel but also to practicing good hygiene and improved vaccine uptake among at-risk groups [46]. However, some regions reported an increased hepatitis A occurrence during the COVID-19 pandemic, e.g., in Bulgaria, when the prevalence of HAV infections among patients hospitalized with viral hepatitis was approximately two-fold higher between May 2020 and April 2021 than in the two preceding years [47]. There are also some reports indicating that increasing HAV susceptibility is correlated with increased COVID-19 severity [48]. Therefore, it is plausible that a relatively stable prevalence of HAV infections during the COVID-19 pandemic in Poland, also encompassing periods of strict sanitary restrictions, is, at least to some extent, a by-product of increased SARS-CoV-2 surveillance in patients revealing various symptoms, including liver-related symptoms.

Notably, the rate of HAV infection increased during the period dominated by the Omicron SARS-CoV-2 lineage, compared to the preceding pandemic waves. This phenomenon can be explained two-fold. Firstly, in February 2022, a war in neighboring Ukraine began, leading to the largest number of displaced people since the Second World War and massive refugee inflow [49]. Within only the first month of the war, 1.8 million people crossed the Polish–Ukrainian border, while, a year later, 1.4 million Ukrainian citizens had a valid residence permit in Poland, predominantly women and children [50]. Before the war, Ukraine was a low-endemicity zone in urban areas and an intermediate-endemicity zone in rural regions [10,51]. However, the movement of refugees in response to war, coupled with the high number of susceptible individuals among children and adolescents in Ukraine, due to low vaccination rates and deteriorated sanitary conditions while in transit, posed a risk of HAV spread [52]. In addition, in Western Ukraine, the incidence of hepatitis A was reported to exceed the country’s average level, a phenomenon attributed to technological challenges in water supply, sewage networks, and wastewater treatment [53]. Our data indicates that the inflow of refugees likely contributed to newly diagnosed HAV infections in Poland since the rate of HAV infections systematically increased from February to August 2022—by 440%. One should also note that the Ministry of Health of Ukraine does not list HAV vaccinations among the recommended vaccinations [54], and interest in receiving such a vaccine is also very low among war refugees—between 24 February 2022 and 30 January 2024, HAV vaccination was received by only 123 individuals of over 2 million Ukrainian citizens who had a valid residence permit (resulting in a rate of 0.06 per 1000, which is 13-fold lower than the average vaccination rate among the inhabitants of Poland in 2022 [55]).

However, one should note that according to the Regulation of the Council of Ministers of 25 March 2022 on establishing specific restrictions, orders, and prohibitions in relation to the state of the epidemic from 28 March 2022 onward, all restrictions related to the COVID-19 pandemic were lifted in Poland [56]. This decision was made despite the fact that it took another 13 months for the WHO to announce that COVID-19 has the status of a Public Health Emergency of International Concern [57]. Therefore, it is likely that a notable spike in HAV infections was a joint result of lifted restrictions and the associated changes to social behaviors and access to healthcare services (including diagnostics), as well as an inflow of imported cases from Ukraine.

Importantly, during the later phase of the Omicron lineage dominance, the rate of newly diagnosed HAV infections was higher than in the pre-Delta and Delta phases and exceeded five-fold that found between early 2014 and early 2017. This rate remained higher after May 2023, when the WHO announced the end of the Public Health Emergency of International Concern (PHEIC) for COVID-19, indicating that HAV spread has reached a relatively stable level in Poland. This observation implies the need to consider vaccination programs targeting at-risk groups, including MSM and individuals with chronic liver disease.

We wish to stress the limitations of this study. It did not include information on sexual orientation or nationality, due to the lack of data. Therefore, the discussed associations of hepatitis A occurrence in MSM and in Ukrainian war refugees must be treated with caution but also considered a motivation for further epidemiological studies focusing on HAV in Poland. This is especially important if one considers that the war-related crisis in Ukraine has led to internal HAV outbreaks in 2023 [58,59].

## 5. Conclusions

The present study is the most recent epidemiological assessment of HAV in Poland and can serve as a reference point for further studies encompassing the Central European region. The prevalence of hepatitis A in the investigated period was consistently more common in men and among the inhabitants of urban areas. During the COVID-19 pandemic, the prevalence of HAV infections remained relatively stable, with a noticeable, though modest, spike after the war in Ukraine began and a massive inflow of migrants occurred. Based on the results of the prevalence of HAV infections after Polish authorities lifted the COVID-19 pandemic restriction and the WHO ended the PHEIC declaration, it can be predicted that the epidemiological situation is unlikely to escalate in Poland unless other circumstances arise. However, the situation requires further monitoring, especially if one considers that the war crisis and the related destruction of water and sanitary infrastructure in neighboring Ukraine are increasing the risk of HAV outbreaks. It is reasonable to promote vaccination in migrant groups as well as at-risk groups to attenuate the transmission and decrease the incidence of the severe sequelae of HAV infection.

## Figures and Tables

**Table 1:** General characteristics of hepatitis A virus infections in relation to gender, age, and place of residence over the 2009–2022 period in Poland.

	Rates of Newly Diagnosed Infection per 100,000 Population						
Indicator	Men			Women			*p*-Value
	Median	Minimum	Maximum	Median	Minimum	Maximum	
Total	0.30	0.11	12.55	0.24	0.08	3.39	**0.001**
≤14	0.35	0.07	3.87	0.26	0.04	3.43	0.397
15–24	0.34	0.04	20.08	0.39	0.00	6.23	0.055
25–34	0.43	0.09	31.26	0.37	0.10	5.52	0.198
35–44	0.37	0.10	18.66	0.30	0.07	4.54	**0.009**
45–54	0.20	0.13	9.13	0.23	0.08	4.29	0.140
≥55	0.19	0.02	2.26	0.12	0.02	1.21	**0.048**
Urban areas	0.39	0.13	18.07	0.29	0.07	4.27	**0.002**
Rural areas	0.21	0.07	4.63	0.14	0.07	1.99	**0.009**

**Table 2:** Trends of new HAV infection in men and women in different age groups in Poland over the years 2009–2022.

Indicator	Joinpoint Analysis to Identify Changes in Trends Over the Years 2009–2022						AAPC
	Trend for Period 1	APC	Trend for Period 2	APC	Trend for Period 3	APC	
**MEN**							
Total	2009–2014	−47.4 * **(−71.0, −4.3)**	2014–2017	312.2 (−71.5, 5865.8)	2017–2022	−39.2 (−66.5, 10.6)	6.6 (−15.0, 33.5)
age ≤ 14	2009–2011	−83.4 (−99.8, 921.4)	2011–2018	68.3 (−16.1, 237.5)	2018–2022	−27.7 (−80.3, 165.6)	13.7 (−6.8, 38.8)
age 15–24	2009–2013	−66.9 (−89.2, 1.6)	2013–2017	240.4 (−42.2, 1906.4)	2017–2022	−38.7 (−72.3, 35.5)	8.1 (−16.8, 40.4)
age 25–34	2009–2014	−54.9 * **(−77.7, −8.6)**	2014–2017	388.1 (−79.1, 11,312.4)	2017–2022	−46.2 (−73.4, 8.9)	2.3 (−21.6, 33.6)
age 35–44	2009–2015	−37.3 * **(−59.1, −3.9)**	2015–2018	326.3 (−66.0, 5239.8)	2018–2022	−60.9 * **(−82.4, 13.0)**	1.7 (−19.1, 28.0)
age 45–54	2009–2015	−27.9 (−54.3, 13.8)	2015–2018	319.5 (−71.8, 6140.9)	2018–2022	−65.3 * **(−85.2, −18.4)**	4.7 (−16.2, 40.7)
age ≥ 55	2009–2013	−49.8 (−76.4, 6.9)	2013–2017	197.6 (−10.0, 883.4)	2017–2022	−23.1 (−54.9, 31.3)	22.1 * **(1.0, 47.5)**
Urban areas	2009–2014	−49.8 * **(−70.9, −13.5)**	2014–2017	350.7 (−60.6, 5052.7)	2017–2022	−42.0 (−66.3, 0.1)	6.1 (−15.9, 33.9)
Rural areas	2009–2015	−30.1 (−62.5, 30.6)	2015–2018	267.1 (−90.9, 14,655.4)	2018–2022	−50.1 (−84.5, 60.5)	8.1 (−12.4, 33.5)
**WOMEN**							
Total	2009–2015	−25.1 (−53.4, 20.5)	2015–2018	237.3 (−79.7, 5496.4)	2018–2022	−49.1 (−79.1, 23.6)	9.1 (−9.3, 31.2)
age ≤ 14	2009–2015	−39.5 (−63.9, 1.4)	2015–2018	374.7 (−77.7, 9998.2)	2018–2022	−43.3 (−78.5, 49.0)	13.2 (−9.6, 41.8)
age 15–24	2009–2015	−31.0 (−64.3, 33.0)	2015–2018	360.9 (−90.5, 22,372.7)	2018–2020	−81.4 (−99.6, 809.2)	11.7 (−16.5, 49.4) †
age 25–34	2009–2015	−23.7 (−55.1, 29.7)	2015–2018	229.8 (−86.1, 7258.2)	2018–2022	−48.8 (−81.0, 38.1)	8.4 (−10.3, 30.9)
age 35–44	2009–2013	−46.3 (−82.6, 66.3)	2013–2018	119.5 (−29.1, 579.3)	2018–2022	−49.9 (−83.8, 55.1)	9.3 (−9.7, 32.2)
age 45–54	2009–2015	−9.7 (−46.2, 51.4)	2015–2018	227.1 (−84.7, 6878.7)	2018–2022	−53.7 (−82.4, 21.7)	14.8 (−5.4, 39.3)
age ≥ 55	2009–2015	−22.3 (−48.5, 17.2)	2015–2018	243.6 (−69.8, 3815.8)	2018–2022	−38.4 (−71.5, 32.9)	16.8 (−2.1, 39.3)
Urban areas	2009–2015	−27.1 (−52.6, 12.3)	2015–2018	263.7 (−71.7, 4576.9)	2018–2022	−51.4 (−78.3, 9.0)	9.2 (−10.0, 32.3)
Rural areas	2009–2015	−23.3 (−58.0, 40.0)	2015–2018	200.9 (−91.4, 10,466.8)	2018–2022	−43.8 (−81.8, 73.0)	9.0 (−9.0, 30.6)

APC—annual percentage change, AAPC—average annual percentage change, HAV—hepatitis A virus; *—statistically significant trend at *p* < 0.05 (in bold); †—AAPC over the years 2009–2020.

## Data Availability

Data were collected from publicly archived datasets of the Epimeld database published by the National Institute of Public Health-National Research Institute (NIPH-NRI) and the Chief Sanitary Inspectorate in Poland.
